# Customizing Starch Properties: A Review of Starch Modifications and Their Applications

**DOI:** 10.3390/polym15163491

**Published:** 2023-08-21

**Authors:** Julia Compart, Aakanksha Singh, Joerg Fettke, Ardha Apriyanto

**Affiliations:** Biopolymer Analytics, Institute of Biochemistry and Biology, University of Potsdam, Karl-Liebknecht-Str. 24-25, Building 20, Golm, 14476 Potsdam, Germany; compart@uni-potsdam.de (J.C.); singh2@uni-potsdam.de (A.S.); apriyanto@uni-potsdam.de (A.A.)

**Keywords:** starch, starch modification, *in planta* modification, physical modification, chemical modification, enzymatic modification, starch application

## Abstract

Starch has been a convenient, economically important polymer with substantial applications in the food and processing industry. However, native starches present restricted applications, which hinder their industrial usage. Therefore, modification of starch is carried out to augment the positive characteristics and eliminate the limitations of the native starches. Modifications of starch can result in generating novel polymers with numerous functional and value-added properties that suit the needs of the industry. Here, we summarize the possible starch modifications *in planta* and outside the plant system (physical, chemical, and enzymatic) and their corresponding applications. In addition, this review will highlight the implications of each starch property adjustment.

## 1. Introduction

Starch is an essential biopolymer found abundantly in nature. It is the main energy and nutrient source of plants to overcome periods where photosynthesis is not possible but also for heterotrophic species such as humans and animals. Furthermore, starch is not only a source of dietary carbohydrates but also an ubiquitous raw material commonly utilized in non-food industry. In principle two types of starch exist, storage starch and transitory starch. The latter is synthesized in chloroplasts of leaves and followed a diurnal cycle of synthesis and degradation. By contrast, storage starch is synthesized in non-photosynthetic active plastids, such as amyloplasts in seeds or storage organs and remains unmetabolized for a longer time period until it is degraded once to support regrowth/sprouting or germination. Typically, the amount of storage starch is much higher than that of transitory starch and therefore this type of starch is commercially used. Compared to other polymers, starch is a multifunctional biomaterial with great potential due to its enormous amount, low cost, and non-toxic properties. Starch is biosynthesized as semi-crystalline granules and is composed of two glucans amylose and amylopectin. Chemically, both homopolysaccharides consist of α-D-glucose units, whereby the linear chains formed by α-1,4-glycosidic linkages and the branching points are introduced by α-1,6 glycosidic bonds [1]. Amylopectin constitutes 70–85% of the starch, is much larger (10^7^–10^9^ Da), and highly branched in comparison to amylose (10^5^–10^6^ Da). However, the ratio of amylose and amylopectin in any native starch varies with the plant genotype.

Moreover, amylopectin is responsible for the semicrystalline structure of the starch granules. The branching points of the amylopectin molecules are clustered, so neighboring glucan chains form double helices, creating crystalline lamellae. The crystalline lamellae alternate with amorphous regions where the branching points are located [2]. The diameter of the starch granules generally ranges from less than 1 µm to more than 100 µm, and the granule shapes also vary from spherical, oval, discoid, polygonal, angular, to quite irregular. In addition, the shape, size, structure, and composition of starch granules are dependent on tissue/organ and the origin of plants [1]. However, independent of the size and morphology, native starch granules exhibit some unfavorable characteristics such as poor solubility, retrogradation, syneresis, thermal decomposition, and high viscosity after gelatinization that limits their industrial usage. Therefore, to broaden the application range of this biopolymer, there is a need for the development and establishment of various starch modification techniques.

During the last decade, the modified starch market has constantly been growing. According to Markets data, the worldwide modified starch trade is expected to be valued at USD 13.7 billion in 2022 and set to gain USD 15.9 billion by 2027, recording a compound annual growth rate (CAGR) of 3.1% (https://www.marketsandmarkets.com/Market-Reports/modified-starch-market-511.html, accessed on 15 August 2023). In addition, the demand for processed and convenience foods is lifting the need for modified starch. Therefore, the usage of modified starch across numerous sectors is expanding as innovative experiments and new technologies in many industrial sectors (food and non-food) progress. Furthermore, due to its high economic and social impact on human life, the starch modification area will be more appealing in the future. Generally, modifications may be divided into two groups, the *in planta* modifications, which can be achieved by genetic manipulation via breeding and molecular biology technologies. The second group includes all modifications outside the plant system that can be obtained by physical, chemical, and/or enzymatic methods. These modifications will be explained in more detail in the following sections.

## 2. Native Starch Modification

It is commonly observed that isolated starch besides amylose and amylopectin also contains minor components such as proteins and lipids [3]. Those can be categorized as non-covalent modifications of starch or simply detected as a contaminant. Until now, phosphoesterification is the only natural covalent modification of starch inside the plant, and it seems that the amylopectins are exclusively phosphorylated [4,5,6]. It was also shown that the phosphate amounts of starches from different tissues, organs, developmental stages, and species are highly variable (As reviewed in [1]). So, it has been reported that the presence of phosphates in the starch granules can affect the degree of crystallinity. The amount of starch phosphoesters in potato tubers have been found to decrease with increasing granule size at both C3- and C6-position [7].

The phosphate content strongly influences the physicochemical properties of starch, such as hydrophilicity, surface charge, chemical vulnerability, crystallinity, and, consequently, influence also properties such as pasting, thermal stability, viscosity, digestibility, and swelling power. Therefore, further elucidating the molecular mechanism of starch phosphorylation is of great interest.

Native starch granules are primarily inert and water-insoluble; thus, alterations of starch characteristics are essential. There are various procedures *in planta* and outside of the plant system to adjust these starch parameters. The aim is always to increase the positive characteristics and/or decrease their defects. *In planta* manipulations are dependent on changes in direct or indirect starch metabolic enzymes. In contrast, outside the plant system modifications include changing the properties of native starches via physical, chemical, and/or enzymatic methods. However, all modifications independent of the manner are strongly linked with a detailed analytical characterization of the starches.

## 3. *In Planta* Starch Modifications

The generation of *in planta* starch modification is the most cost-effective option when specific starch properties for certain applications are needed [8]. Recent discoveries of genes involved in starch metabolism have provided a new route for starch modification inside plants. Starch amount, granule morphology, granule size, inner granule structure, and phosphorylation are starch parameters that can be successfully modified inside the plant. Mutants, which have become extensively used in the food sector for their unique and natural traits, can also be utilized to identify new starch functions. Even though current research primarily uses plant models such as *Arabidopsis thaliana* [1], more research on starch metabolism in other plants, especially starch producing crops, is getting established, so that it is already possible to change also starch parameters in crops by exploiting biotechnological tools (see Table 1). Interestingly, in higher plants, it seems the starch metabolism is greatly evolutionarily conserved so that it is much easier to transfer information from model species to crop-producing starch species [9,10,11].

Genetic modifications avail themselves of the advantage over environmentally hazardous post-harvest chemical, physical and enzymatic modifications and demonstrate great potential. However, its application depends greatly on political decisions since the usage of genetic technologies, e.g., CRISPR-Cas, is still under discussion in many countries.

Unfortunately, no exact single gene mentioned above can regulate a single starch parameter. The genes usually have additional effects on other starch parameters. Due to its complexity, extensive research is still underway to find new genes that regulate the starch properties inside plants. For more information regarding *in planta* starch modifications, see [1].

## 4. Starch Modifications Outside the Plant System

In addition to the above *in planta* starch alterations, modifications outside of the plant are a substantial field, allowing starches to be adapted by physical, chemical, and enzymatic means. The goal is to overcome native starches’ adverse physicochemical features, such as syneresis, retrogradation, and lack of solubility in solvents. In some cases, one type of modification does not satisfy the requirements of industry and/or research, so combining different techniques is unavoidable. However, the first step is always to extract native starch granules from the plant. No universal isolation method exists because many different extraction protocols have been established depending on the plant material. However, all protocols are based on the homogenization of the plant material to break down the cell walls and release the cell contents [37]. A modification of starches during isolation is observed and primarily intended. According to the method of starch extraction and the use of physical forces, chemicals, enzymes, etc., the starch properties can differ (see also below) [38].

### 4.1. Chemical Modifications of Isolated Starches

**Figure 1:**
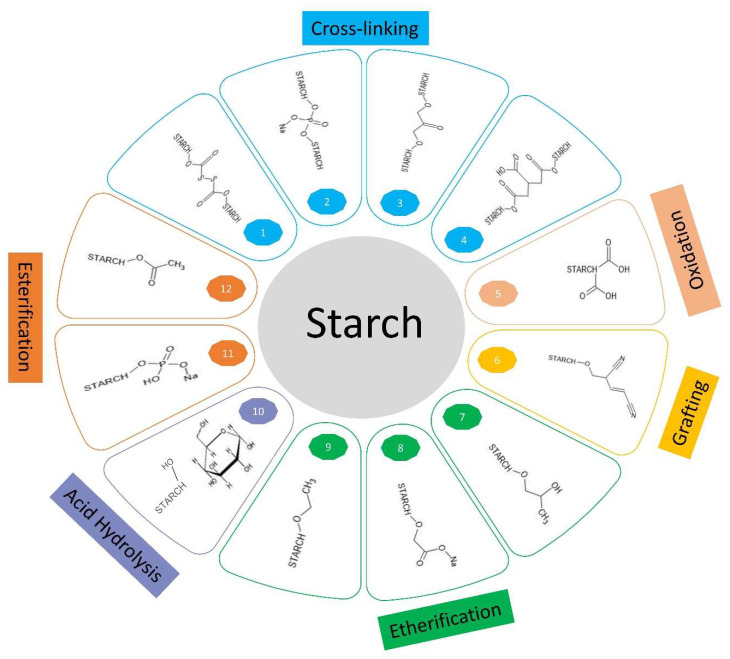
Chemical modification on starch. Cross-linking modification with (1) CS_2_ (2) POCl_3_, Na_3_P_3_O_9_ (3) Epichlorhydrin and (4) C_6_H_5_O(COOH)_3_; Oxidation modification with (5) O_3_, HIO_4_; Grafting modification with (6) acrylamide with possible initiators: CE, (NH_4_)_2_S_2_O_8_, KMnO_$_/HIO_4_; Etherification modification with (7) C_3_H_6_O (8) CH_2_ClCOONa (9) C_2_H_5_Cl; Acid hydrolysis with (10) HCl, TFA, HNO_3_; Esterification modification with (11) Na_2_HPO_4_ (12) CH_3_COOH.

In comparison to physical or enzymatic modifications, chemical techniques enable a much wider spectrum for the functionalization of starch and expand the field of applications. Chemical modifications are non-destructive, and the hydroxyl groups of starches serve as active sites for the introduction of functional groups such as, e.g., acetyl-, carboxyl-, and ethyl-groups which act as electrophilic reactants. However, the rate and efficiency of modification depends on several factors such as starch source, amylose-to-amylopectin ratio, the granule morphology, reaction conditions and the nature and amount of the modifying reagent [39]. The modifications can be categorized into the following classes: cross-linking, esterification, etherification, oxidation, grafting, and acid hydrolysis (Table 2; Figure 1).

Cross-linking is the most frequently applied method for changing starch properties and is used in the pharmaceutical and food industries, wastewater processing, and bioplastic production. Depending on the field of application, several reagents are utilized, such as, e.g., epichlorohydrin (EPI), phosphoryl chloride, adipic acid mixed with anhydride and sodium tripolyphosphate. Cross linking involves the molecular reaction between the reactive hydroxyl groups on starch with multifunctional reagents resulting in ether or ester linkages. Such interactions not only take place between single chains but also links side by side chains as well [40]. As a result of cross-ligation in starch the degree of polymerization, molecular mass and the solubility in organic solvents are enhanced. These interactions resulting from cross-linking can also stabilize and strengthen the granules, especially by enhancing the existing hydrogen bonds through the introduction of additional covalent bonds. Therefore, modified starch exhibits a higher tolerance to acids, heat, and shear forces. Moreover, improved characteristics such as a decrease in retrogradation, a higher viscosity, and better paste clarity are obtained [40,41]. Various factors such as starch parameters, cross linking reagent concentration and composition, pH, reaction time as well as temperature have been found to affect the cross-linking efficiency [42,43].

Esterification takes place on hydroxyl groups at the C3, C2 and C6 positions (order indicates increasing reactivity) [44], whereby two types of esters can be distinguished, organic and inorganic starch esters. It involves the acid-catalyzed mediated substitution reaction of a nucleophilic acyl compound with a molecule containing an acid anhydride, acid chloride, or carboxylic acid structure. With the esterification of hydroxyl groups of available glucose units, the hydrogen bonding ability of amylose or amylopectin is compromised [45,46]. Improved hydrophobicity, swelling capacity, and lower retrogradation rate were observed [47]. Succinylation, as an example for organic esterification reduces the gelatinization temperature and retrogradation while simultaneously increasing the thickening power, viscosity, and water retention properties [48]. An example of an inorganic ester is the product of phosphorylation, as mentioned above. Besides being a natural modification, it is also used industrially to increase the viscosity, pasting transparency, and gelatinization characteristics [49]. However, the rate of esterification greatly depends on the structure of the reacting acids particularly on the steric, mesomeric and inductive effects exerted by them. Similarly other factors such as molar ratio, temperature, reaction duration is equally crucial to determine the effectiveness of the esterification reaction [46]. The degree of substitution (DS) acts as an indicator of quality and physical properties of the starch esters and determines the extent to which the starch can recrystallize or retrograde [50]. The esterification is widely used in paper production, pharmaceuticals, but also in food processing.

Improved properties regarding thermal stability, rheological behavior, and, with restrictions, ionic activity are observed for etherified starch. The etherification substitutes hydroxyl groups with anhydroglucose units using functional groups, which can be positively or negatively charged. Moreover, the generation of amphoteric or non-ionic starches are possible. One important example of a negatively charged starch ether is carboxymethyl starch, classified as a green polymer that covers a broad spectrum of applications in industry, due to its great hydrophilicity and therefore, cold-water solubility, as well as flocculation behavior, for example, in pharmaceutical approaches as a drug delivery system or in waste water purification as biosorbents to sequestrate heavy metals [45,51].

Another opportunity for modifying starch is oxidation, which can cause depolymerization of starch, whereby it enhances hydrophilicity, disrupts molecule linearity and crystallinity, and reduces retrogradation as well as enthalpy by the formation of carbonyl and carboxyl groups in native starch by the use of an oxidizing agent [52]. There are various oxidants available, such as potassium permanganate, sodium hypochlorite, hydrogen peroxide, etc. Oxidation mainly happens at hydroxyl groups C2, C3, and C6, depending on the pH, temperature, and reagent, and results in the formation of dialdehyde- and dicarboxylic acid derivates [45]. Nowadays, the impact of ozone on oxidation reactions has grown, as it can react with starch without any catalyst or special reaction conditions, while additionally exhibiting minimal effects on morphology, crystallinity, and the inner structure, as it acts especially on the amorphous regions of starch [52]. This kind of modification is used for food, textile, and paper processing.

During grafting, a polymer chain (grafting onto) or monomers such as vinyl units (grafting from) is introduced into the starch, resulting in copolymerization that combines the features of both polymers. The initiation of the copolymerization process occurs in particularly at the C1–C2 end groups and the C2–C3 position in the presence of a free radical initiator [45,53]. Therefore, two types of radical initiating can be distinguished: chemical initiation with, e.g., ceric ammonium nitrate or potassium permanganate, or irradiation initiation via UV light or microwaves. It has been reported that the ceric salts form complexes with alcohols and glycols, through disproportionation reaction which is also the rate-determining step of the oxidation-reduction reaction. The key characteristic of the oxidation with ceric ions is that it advances with single electron transfer leading to generation of free radicals on the starch. The presence of free radicals on the starch causes initialization of polymerization to form a graft copolymer [54]. When manganese ions are used for the initiation process, different primary radical species are produced depending on the type and nature of the acid [55]. Only the polar bonds are “selectively excited” by microwave radiation, which results in their cleavage, generating multiple free radicals. The relatively non-polar C-C bonds however remain unaffected [56,57]. Depending on the polymer introduced, the resulting starch reveals different characteristics; mostly, it has higher biodegradability, thermal stability, hydrodynamic radius, and improved flocculation properties [58]. However, the use of microwaves and UV light for graft polymerization in starch has gained momentum in the past few decades because of the environmentally friendly nature and improved properties for commercial utilization. Grafting is mainly used in electrical engineering, production of bioplastics, agriculture as well as in pharmacy.

**Table 2:** Chemical modifications of starch.

Modification	Implications	Industrial Sectors	Example Applications	References
**Cross-linking** (Formation of inter and intramolecular bridges resulting from an interaction between reactive hydroxyl groups in starch and reagents.)	Higher stability of granules towards swelling, high temperature, and high shear and acidic conditions	Food	Viscosifiers and texturizers in dairy products	[40]
		Pharmacy	Transport of molecules and excipients	[40]
		Wastewater treatment	Chelation of pollutants	
		Packaging	Bioplastics	
**Esterification** (Condensation of the carboxylic acids, fatty acids or phosphates with reactive hydroxyl groups of the starch)	Lower gelatinization temperature and retrogradation, lower tendency to form gels, and higher paste clarity	Food	Emulsion stabilizer in refrigerated and frozen foods	[50,59]
		Textiles	Film-forming polymer	
		Paper production	For packaging	
**Etherification** (Substitution of the reactive hydroxyl groups with anhydroglucose units using positively or negatively charged functional groups)	Improves the clarity of starch pastes, increases viscosity, reduces syneresis, and increases freeze-thaw stability	Pharmacy	Drug delivery	[60,61]
		Wastewater treatment	Adsorbents of heavy metal ions	
**Oxidation** (Involves oxidation of primary or secondary hydroxyl groups of the glucose units of starch with formation of carbonyl or carboxyl groups using various oxidizing agents)	Low viscosity, high clarity, and low-temperature stability	Food	In batter and bread for coating various foodstuffs and film formers,	[62,63]
		Confection dairy	As binders and texturizers	
		Paper Textiles	Improved strength and printability	
**Grafting** (Grafting of acrylic monomers onto the starch via free radicals generated through different free radical initiators)	High viscosity, thermal stability, biodegradability	Cosmetics	Moisturizer, skin-and hair products, perfumes	[58]
		Pharmacy	Microparticle system for vaccine delivery	
		Agriculture	For mulching and controlling weeds, conserves soil moisture and heat	
		Electrical engineering	Cable sealing	
		Wastewater treatment	heavy metal ion removal	
		Bioplastic	biodegradable plastics and films	
**Acid hydrolysis** (Hydrolysis of the glycosidic linkage because of attack of the hydronium ion on the oxygen atom in the glycosidic bond)	Low paste viscosity, high gel strength and water solubility	Food	Gelling agent in the production of gum and processed cheese loaves, fat replacers/fat mimetic	[64,65]

The acid hydrolysis of starch is performed at temperatures below the gelatinization temperature and favors properties such as solubility, pasting viscosity, gelatinization enthalpy, and swelling power, whereas the effect on granular morphology differs among species, tissues, and degrees of hydrolysis. For instance, a slight hydrolysis reveals no significant morphological alteration, which presupposes that the starch has not been boiled [66]. In general, the glycosidic bond can be attacked by various acids. The most common is the use of nitric, sulfuric, or phosphoric acid. The acid acts primarily on the starch granule surface before entering the inner structure [67]. This modification technique is mainly implemented in the food, paper, and textile industries.

### 4.2. Physical Modifications of Isolated Starches

Generally, physical modifications can be used to interrupt or alter the granular size and/or packing arrangement. Thus, physical modifications can generate starch with similar characteristics as those obtained from chemical treatments, but without any agent which is toxic or must be removed thereafter. The methods are easy, cost-effective, and largely environmentally friendly (Table 3). Overall, physical modifications can be divided into two groups: thermal and non-thermal methods. The main thermal modifications include pregelatinization, extrusion, heat-moisturizing, annealing, and microwave treatments. Applications such as ultra-high pressure, ultrasonification, and pulse electric field are amongst the non-thermal methods.

Pregelatinization is a very simple modification that includes the boiling of starch in an aqueous solution followed by drum or spray drying, which then results in reduced hydrogen bonding, fragmentation, and the molecular disintegration of starch granules. The spray dried gelatinized starch finds its application in the microcapsule industry for the purpose of drug release due to its low cost and availability [68]. The pregelatinized starch is cold water soluble, shows no optical birefringence, and has enhanced viscosity and gel stability [49,69].

Extrusion is a thermomechanical method involving continuously forcing gelatinized starch under pressure out of a shaping aperture. This technique plays a role in the packaging and food industry [70]. However, small alterations in processing conditions can influence the quality and properties of extrudates. For instance, when rice flour is extruded under low-expansion condition recombined rice is obtained whereas under high-expansion conditions crispy rice is produced [71].

The most practiced thermal methods are heat-moisture treatment (HMT) and annealing. The physicochemical properties of starch are greatly enhanced by both of these hydrothermal processes without affecting the granular structure. Both methods differ in the moisture content used. While annealing employs an excessive amount of water (>40%), a long processing time, and temperatures over the glass transition but below the gelatinization temperature, HMT requires a high temperature and a low moisture content range (10–35%). HMT alters the crystalline and amorphous layers of granules and thus reduces swelling property, solubility, and viscosity, simultaneously raising the pasting temperature. However, the efficiency of this technique greatly depends upon the process variables and the source of starch. The lower the moisture used, the greater is the alteration on the starch granule surface and hence increased susceptibility to other types of modifications. Additionally, starch derived from cereals shows lesser sensitivity to alterations than the tuber and root starches [72].

Annealing improves the crystallinity by providing molecular rearrangement and lowering the free energy; thus, more organized structures can form [73]. Since the heat required for gelatinization of starch is inversely correlated to the area of the starch crystalline region; thus, it is a method that works better with starches that revealed increased amorphous regions. Due to rearrangement of the crystalline structure, starch presents greater resistance to digestibility after application of annealing technology [73].

Microwaves are electromagnetic waves with a wavelength between 1 mm and 1 m corresponding to the frequency range between 300 MHz and 300 GHz [74]. Polar molecules (e.g., water molecules) absorb microwave energy, rapidly align themselves in response to the electric field, and produce bulk heat by molecular friction. Microwave treatment of starch resulted in lower melting enthalpy, swelling strength, and solubility, as well as higher gelatinization temperatures [39,41], which is particularly relevant in the pharmaceutical industry.

Micronization is a significant reduction of the average particle size. It can be a thermal treatment with infrared radiation in the range of 1800 to 3400 nm. It also heats starch in a short amount of time [75]. Non-thermal treatments include the use of a vacuum ball-mill or a hammer- or pin mill, which utilize mechanical friction forces and compression to break down granules into fragments or fine powder. Crystallinity and molecular structures are destroyed in addition to granule morphology. Cryogenic micronization, a much gentler technique, has been used for cereal grains. It causes marginal damage of the starch structure but it has not yet been applied on an industrial scale, especially in the food industry [76].

Another technology used for physical modification is the γ-ray irradiation treatment of starch. Plenty of research has been done in the past few decades to assess the impact of γ-radiation on starch granules. Radiation processing via γ-irradiation includes the utilization of a radioactive isotope, either in the form of cesium 137 or cobalt-60. These isotopes can emit high energy γ-rays or photons and are capable of invading great depth into the target product. The γ-irradiation causes a decrease in crystallinity, the swelling index, and pasting properties. In addition, starch granules become internally cracked, but surface cracking is not observed [77].Factors such as water content of the samples, type of gas atmosphere, dose and rate of dose as well as structure and composition of starch affects the impact of γ-irradiation. However, this method needs safety considerations.

Furthermore, starch can be exposed to ultra-high pressures (100–1000 MPa). High pressure is a green and eco-friendly technique capable of altering non-covalent chemical linkages with very minor effects on the covalent linkages. Depending on duration, pressure intensity, temperature, and starch origin, the swelling power and solubility increase and retrogradation is delayed. Moreover, the thermal and pasting properties, as well as the amounts of resistant, fast, and slow digestible starch, are altered significantly [78]. Ultra-high pressure is applied for food processing.

The application of frequencies above 20 kHz to agitate particles is known as ultrasonication. The sonication results in acoustic cavitation which is characterized by the rapid development and dissolution of bubbles in a liquid exposed to heat and pressure. The effects of ultrasonic treatment on starch properties depend on the time, intensity, frequency, type, and structure of the starch. However, it has been demonstrated that ultra sonification generates cracks and cavities on the granule surface and thereby increases the water-solubility and rheological characteristics [79]. This kind of modification is important to produce biofuels as well as for pharmaceutical applications as preparation of nanoparticles for drug delivery.

**Table 3:** Physical modifications of starch.

Modification Method	Implications	Industrial Sectors	Example Applications	References
**Microwaves** (Starch modification technique that involves usage of electromagnetic waves in the frequency range of 300–300,000 MHz to generate heat with the help of “molecular friction” in the presence of alternating electric field)	Change the morphology, crystallinity, the gelatinization mechanism, and the rheological properties of the starch	Pharmacy	Control of drug release	[80,81]
**Ultrasonic** (Involves utilization of frequencies above 20 kHz to modify starch by mechanical and cavitation effect)	Modifies the swelling of granules and pastes	Pharmacy	Reduction of pathogens; Preparation of nanoparticles	[82,83,84]
		Energy	Pretreatment for production of biofuel	
		Wastewater treatment	Recycling industrial waste containing starch	
**Ultra-high pressure** (A non-thermal technique capable of altering non-covalent chemical linkages by the application of ultra-high pressures (100–1000 MPa)	Reduces the swelling and viscosity of starch, lower gelatinization temperature	Food	Applied in the binding of aroma compounds; textural improvement, sterilizing of food	[85,86]
**Extrusion** (A thermomechanical procedure that can rupture the starch bonds leading to its degradation and gelatinization)	Breakdown of granules and destroying crystallinity, increase of water absorption	Engineered food	Meat analogous	[87]
		Paper/packaging	Bioplastics	
**Heat moisture treatment** (A hydrothermal treatment that involves heating starch granules for a fixed period of time under low moisture conditions and at temperatures above the glass transition temperature but below the gelatinization temperature)	Increase thermal transition temperatures (onset temperature (To), peak temperature (Tp), and conclusion temperature (Tc)	Food	Production of retort foods, dressings, noodles, baked foods, batter products, confections, dairy products, creams, fat mimetics, and resistant starches	[88]
**Annealing** (A hydrothermal modification method that involves heating of starch granules under high moisture conditions, between their glass transition and gelatinization temperatures for extended period)	Increase gelatinization transition temperature, crystallinity and heat stability	Food	Improve texture for bread making; canned and frozen food processing; improving noodles quality	[89]
**Micronization** (It involves utilization of infrared technology to heat and vibrate starch molecules resulting in increased gelatinization)	Destroying of granules and interrupting crystallinity, increase of solubility, decrease of swelling property, reduction of gelatinization transition temperature and enthalpy	Food	Fat substitute, noodles, convenience, and fried food	[89]
**γ-irradiation** (An ionizing and non-thermal physical method involving the use of a radioactive isotope that emits high-energy—γ-rays or photons capable of intruding in-depth into the starch)	Swelling power and viscosity decreased, reduction of viscosity and gelatinization enthalpy, high water solubility	Food	Preparation of water-soluble starch for food application, animal feed production	[90,91,92,93]
**Plasma** (Involves exposure of the starch granules to plasma (ionized gas) resulting in starch structure modification)	Crystallinity reduced, swelling ability and viscosity decreased, alteration of gelatinization temperature and molecular weight	Food	Desinfection/sterilization	[94,95]

Pulse electric field technology, wherein a field strength of around 10–80 kV/cm for a period of micro- or milliseconds is used, can result in split or deformed starch granules. With increasing electric field strength, pasting properties such as the peak, breakdown, and final viscosity drop, and the crystallinity decreases. However, product properties (such as molecular weight, pH, conductivity) and temperature conditions of the reaction system can affect the modification of starch by influencing the pulse electric field intensity. Furthermore, ohmic heating can lead to gelatinization [96,97].

Another innovative non-thermal technology for modifying starch is cold plasma treatment. Plasma is a mixture of ions, radicals, free electrons, and neutral atoms and molecules. Accordingly, the degree of ionization can vary between 1 and 100% and is generated via an energy input through electric or magnetic fields and, radio waves, microwaves, or heat. Among these, an electric field is the most frequently used energy source. Likewise, the effect on starch properties can significantly differ depending on the exposure time, power and volt supply, and the gas composition used, so several modifications are possible, such as cross-linking, depolymerization, and etching. Thus, plasma treatment leads to either an increase or decrease in molecular weight. The same is also valid for the gelatinization temperature. Furthermore, this physical method destroys starch granules’ crystallinity. The degree of moisture content must also be considered because the bombardment with plasma can produce hydroxyl radicals that favor further breakdown of crystalline regions. This can also cause cracks and holes in granules. The use of plasma is a promising technique with numerous applications, as it allows for several types of starch modification. Importantly, it is also fast, simple, and eco-friendly, leaving no chemical and/or toxic waste products. Starch-related plasma research is in its early stages and there is an enormous energy demand that accompanies the process of utilization, so, in the end, a cost-benefit calculation is crucial [94]. This technology is currently used in food processing. It has also been shown to significantly improve seed germination, so it may have potential for industrial use in agriculture in the future [98].

### 4.3. Enzymatic Modifications of Isolated Starches

**Figure 2:**
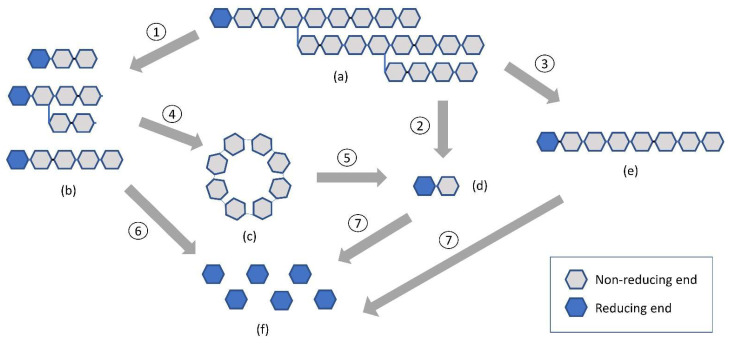
Enzymatic action on starch. (**a**) starch (**b**) α-limit dextrin and simple oligosaccharides (**c**) cyclodextrin (**d**) maltose (**e**) linear oligosaccharides (**f**) glucose. (1) α-amylase [AMY; EC 3.2.1.1]; (2) β-amylase [BAM; EC 3.2.1.2]; (3) Pullulanase [PULA; EC 3.2.1.41] or Isoamylase [ISA; EC 3.2.1.68]; (4) Cyclodextrin glycosyltransferase [CDGT; EC 2.4.1.19]; (5) Cyclomaltodextrinase [CDase; EC 3.2.1.54]; (6) Amyloglucosidase [AMG; EC 3.2.1.3] or α-glucosidase [AGD; EC 3.2.1.20]; (7) Cyclomaltodextrinase or α-glucosidase.

Enzymatic modification is referred to as the utilization of enzymes that alter starch parameters. This kind of modification is primarily applied in the food industry. Traditionally, starch modifications by an enzymatic reaction are generally divided into four categories: endoamylase, exoamylase, debranching enzymes, and transferases (Table 4; Figure 2) However, further potential of various enzymes/proteins was mentioned before.

α-amylases are a well-known example of the endoamylase category. These enzymes randomly hydrolyze starch at any (1,4)-linkage within the two starch polymers, amylose, and amylopectin. As a result, they reduce the chain length [99]. Depending on the type and concentration of enzyme being employed, α-amylase can produce pockets at the surface of starch granules. Exoamylases include β-amylases [100], Amyloglucosidases [101], and α-glucosidases [102]. Exoamylases act on external glucose residues of amylose or amylopectin and thus produce β-limit dextrins (β-amylase), maltose, and glucose (glucoamylase and α-glucosidase) [103]. The enzymatic modification by β-amylases results in the development of multiple cracks on the starch granule surface. Compared to native starch, the enzymatically modified starch exhibits increased solubility but decreased swelling capacity and pasting viscosity.

**Table 4:** Enzymatic modifications of starch.

Modification	Mechanisms	Industrial Sectors	Example Applications	References
**β-amylase** **[BAM; EC 3.2.1.2]**	Cleave α-(1,4) glycosidic bonds from the nonreducing ends of the glucan chains	Food	Producing high-maltose syrups	[104,105]
**Amyloglucosidase** **[AMG; EC 3.2.1.3]**	Cleave both α-(1,4) and α-(1,6) glycosidic bonds from the nonreducing ends of the glucan chains	Food	Production of high-glucose syrups and high-fructose syrups	[106,107]
		Energy	Biofuel production, produce fermentable sugars to produce ethanol	
**α-glucosidase** **[AGD; EC 3.2.1.20]**	Hydrolysis of terminal, nonreducing (1,4) linked α-D-glucose residues with release of α-D-glucose from complex polymers with α-(1,4) bonds, such as malto-oligosaccharides, soluble starch, amylose and glycogen	Food	Production of isomalto- and malto-oligosaccharides with prebiotic activity; production of glucose from starchy sources	[102,108,109,110,111]
		Energy	Biofuel production	
		Pharmacy	Medical biosensors	
**α-amylase** **[AMY; EC 3.2.1.1]**	Endohydrolysis of α-(1,4) glycosidic linkage in polysacharides containing three or more α-(1,4) linked D-glucose units	Food	Saccharification or liquefaction of starch, the clarification of haze formed in beer or fruit juices, and the pretreatment of animal feed to improve digestibility	[39,112,113,114]
		Textiles	Preparation of viscous and stable starch solutions for the sizing of textile fibers	
		Detergent	Improvement of detergency of laundry bleach composition and bleaching without color darkening	
		Energy	Biofuel production, produce fermentable sugars to produce ethanol	
**Maltogenic α-amylase** **[EC 3.2.1.133]**	Hydrolysis of *α*-(1,4) glycosidic linkage in glucan chains to remove alpha-maltose from the non-reducing ends. Maltose production from amylose, amylopectin, and cyclodextrin in exo/or endo-like manner	Food	Prevention of staling of bakery products; non-digestible starch	[115,116]
**Isoamylase** **[ISA; EC 3.2.1.68]**	Hydrolyse the α-(1,6) glucosidic bond in amylopectin, glycogen and their β-limit dextrins. Inability to hydrolize pullulan.	Food	Saccharification or liquefaction of starch	[117]
**Pullulanase** **[PULA; EC 3.2.1.41]**	Hydrolyse the α-(1,6) glucosidic bond in pullulan, amylopectin, glycogen, also in alpha- and beta-limit dextrins of amylopectin and glycogen	Food	Saccharification or liquefaction of starch; production of high-glucose or high-maltose syrups; manufacturing of low-calorie beer; anti-stalling agent to improve texture, volume, and flavor of bakery products	[99,117,118]
		Detergent	Additives in dishwashing and laundry detergents for the removal of starches	
		Paper	Used in adhesive products and in the production of corrugated board and paper	
**Amylomaltase** **[EC 2.4.1.25]**	Transfers a segment of a (1,4)- α-D-glucan to a new position in an acceptor, which may be glucose or α (1,4)-α-D-glucan	Food	Fat replacer and enhancer of creaminess in yoghurt and mayonnaise; production of plant-based alternative to gelatin; prevention of staling of rice cake and bakery products; thermoreversible gel; lump-free cooked rice and porridge	[99,116,119,120,121,122,123]
**Cyclodextrin glycosyltransferase** **[CDGT; EC 2.4.1.19]**	Cyclizes part of a α-(1,4)-D-glucan chain by formation of α-(1,4) glycosidic linkage	Food	Production of cyclodextrins; glycosylation of the potent sweetener stevioside; retard of bread/rice cake retrogradation	[39,124,125,126,127]
		Pharmaceutical	Drug delivery systems	
**Amylosucrase** **[AMYS; EC 2.4.1.4]**	Sequential transglycosylation of glucosyl unit from sucrose onto acceptor molecule	Food	Resistant starch; slowly digestible starch	[128,129,130]

The debranching enzyme category includes isoamylase [99] and pullulanase [131,132]. Debranching enzymes can hydrolyze the α-1,6 glucosidic bond. The major difference between isoamylase and pullulanase is that the pullulanase can hydrolyze the α-1,6 glycosidic bond in starch and pullulan, while isoamylase can only hydrolyze the α-1,6 bond in glycogen and starch [117]. These enzymes produce linear glucans by specifically degrading amylopectin.

The transferases include amylomaltase [99,133], cyclodextrin glycosyltransferase [99,133], and amylosucrase [134]. Transferases hydrolyze the α-1,4 glycosidic bond of a donor molecule and transmit the cleaved residue from the donor to a glycosidic acceptor with the formation of a new glycosidic bond [39].

The hydrolysis rate values for each enzyme mentioned above significantly differ depending on the starch. For native starch, the differences mainly depend on its structural properties [47,135].Since the production of enzymes on an industrial scale, such as through production of amylases by bacteria and fungi, and germination of cereals, the enzymatic modification of starch has gained commercial importance. This technology can be utilized to efficiently manage the specific properties of starch. However, a single enzyme does not substantially alter the properties of starch and therefore combination of enzymes is generally used to achieve the desired results. The specificity of the reaction, which takes place under mild temperature, pH, and agitation conditions, as well as the low effluent generation adds up to the advantages of utilizing enzymes. Furthermore, since the concentration of the enzyme used in the reaction is much lower than that of starch, introduction of even slightest of variations in the reaction medium can interrupt the reaction. These factors make enzymatic modification a very promising and attractive method for starch remodeling. However, due to the time-consuming nature of the enzyme-based method, its application prospects are hindered and therefore, there is a need of using this technology in conjunction with other modification methods. Furthermore, even though there are plenty of available enzyme resources with different properties in microbial or gene databases, the natural enzymes still possess some inevitable defects such as structural instability and low catalytic efficiency due to limitations of biological adaptation and evolution. Therefore, there is a need for improving the properties of industrially important enzymes. This could be achieved through enzyme engineering which involves altering the amino acid sequence of the enzymes and therefore plays a central role in developing efficient biocatalysts with good industrial application potential. In addition to this, attention should be paid to improving the production yields of the enzymes which demand optimization of expression elements and secretory pathways of the host cell and improvisation of high-density fermentation strategies.

Finally, there are numerous possibilities and a great need to modify starch to change the properties in the direction required for the respective application area (see Section 4). Not all the types of modification listed here are also applied in industrial processes (so far), but there is no denying that future technologies will have to be developed/utilized which agree with sustainability, climate, and environmental protection. This involves the environmental safety of the reaction components used, process and production reliability, energy requirements, resource conservation and material recovery. Examples of such new technologies are on the one hand the material use of CO_2_, which is available as a quasi-inexhaustible resource, and on the other hand the use of cold plasma.

The key to success, however, is most likely to be found in the use of interdisciplinary methods; in a combination of chemical/physical methods and the use of enzymes, the reactions can occur under very mild and efficient conditions. One such example is the starch esterification, where a very high degree of substitution of the hydroxyl groups can be achieved by using lipase in combination with microwave irradiation in an ionic solution [136].

## 5. Conclusions

The impact of starch and its utilization has enormously increased in recent years and with it the development of novel technologies that pursue the aim of modifying starch in the best conceivable manner for the respective demands of the application. The precise modulation of starch enhances its industrial applicability. Several ways have been devised to adjust the starch characteristics which can be implemented either inside the plant or outside the plant system (physical, chemical, and enzymatic modifications). However, each method comes with its own set of pros and cons. For example, the modification of starch by means of chemicals is a preferred method due to its ease of modification and low cost of operation. However, in the current scenario, it is not acceptable due to increasing consumer demand for safe, high-quality products as the use of acids, acetates, hypochlorites, phosphates, etc., employed for modification can lead to the generation of chemical wastes that may be detrimental to the ecosystem and requires recycling. Furthermore, the physical modifications on the one hand are mostly eco-friendly but on the other hand, sometimes the alterations in starch structure are not as severe or obvious as those created through chemical modifications. Though enzyme modification methods can be used to effectively control the specific properties of starch, its application is hindered by factors such as low thermostability and catalytic efficiency of the enzymes. Similarly, although the genetic modification of plants offers the potential to generate novel starches which can lessen or eradicate the use of environmentally perilous post-harvest chemical and enzymatic modification, its usage is subject to legislative approval, limiting its global application. Thus, there is an inherent need for the development of integrative methods that are efficient, politically acceptable, inexpensive, as well as environmentally friendly. One such strategy could be the multiple modifications of starch with the desired functional and nutritional properties which employ either combination of several similar methods (homogeneous) or several different methods (heterogeneous). However, the choice of modification types and treatment methods depends on the types of changes in functionality and reactivity of starch that are required for a specific application. Despite some remarkable progress that has been made in starch analytics, time, and spatial resolutions often remain limited. Consequently, establishing and developing new starch analytical techniques are also essential in the future.

## Figures and Tables

**Table 1:** *In planta* modifications of starch.

Starch Parameter	Gene Target	Organism Species (Gene Changes)	Structural Changes	Reference
Granule size	B-Granule Content 1 (BGC1)	Wheat *Triticum aestivum* (mutation)	Reduced B-type granules	[12]
	Floury Endosperm 6 (FLO6)	Barley *Hordeum vulgare* (mutation)	Fractured granules	[13,14]
	Floury Endosperm 6 (FLO6)	Rice *Oryza sativa* (mutation)	Formation of compound granule was defective—smaller irregular granules	[15]
	Substandard Starch Grain 4/6 (SSG4 or SSG6)	Rice *Oryza sativa* (mutation)	Higher compound granule size	[16,17]
	Starch Synthase 1 (SS1)	Sweet potato *Ipomoea batatas* (overexpression)	Higher granule size Bimodal granule size distribution	[18]
Granule morphology	Starch Synthase 3a/4b (SS3a/SS4b)	Rice *Oryza sativa* (mutation)	Change granules from polyhedral to spherical	[19]
	Starch Synthase 4 (SS4)	Arabidopsis *Arabidopsis thaliana* (mutation)	Change granules from discoid to spherical	[20,21]
	Cytosolic disproportionating enzyme 2 (DPE2) and plastidial phosphorylase (PHS1)	Arabidopsis *Arabidopsis thaliana* (mutation)	Change granules from discoid to spherical	[22]
Amylopectin content	Starch Synthase 2 (SS2)	Rice *Oryza sativa* (mutation)	Higher in amylopectin short chains	[23]
		Sweet potato *Ipomoea batatas* (suppression)	Higher in amylopectin short chains	[24]
	Starch Synthase 3 (SS3)	Tomato *Solanum lycopersicum* (overexpression)	Higher amylopectin content	[25]
	Starch Branching Enzyme 2 (SBE2)	Potato *Solanum tuberosum* (overexpression)	Higher in amylopectin short chains, more branches	[26]
Amylose content	Granule-Bound Starch Synthase (GBSS)	Rice *Oryza sativa* (mutation)	Lower amylose content	[27,28]
		Cassava *Manihot esculenta* (mutation)	Lower amylose content	[29]
		Sweet potato *Ipomoea batatas* (suppression)	Lower amylose content Absence of long amylopectin chains	[30]
		Potato *Solanum tuberosum* (suppression)	Lower amylose content	[31]
	Starch Branching Enzyme (SBEIIb)	Rice *Oryza sativa* (mutation)	Higher amylose content Higher in amylopectin long chains Change of granule morphology	[32]
		Maize *Zea mays* (mutation)	Amylose extender starch Higher amylose content Higher in amylopectin long chains Change of granule morphology	[11]
	Starch Branching Enzyme (SBEI/SBEII)	Potato *Solanum tuberosum* (suppression)	Higher amylose content Higher in amylopectin long chains Change of granule morphology	[33]
Phosphate content	Glucan, Water Dikinase (GWD)	Barley *Hordeum vulgare* (overexpression)	Higher phosphate content Change of granule morphology	[34]
		Cassava *Manihot esculenta* (overexpression)	Higher phosphate content	[10]
		Potato *Solanum tuberosum* (suppression)	Lower phosphate content Higher amylose content	[35]
		Rice *Oryza sativa* (overexpression)	Higher phosphate content	[36]
	Starch Excess 4 or Like Sex Four 2 (SEX4 or LSF2)	Potato *Solanum tuberosum* (suppression)	Higher phosphate content Altered amylopectin structure. Reduced granule size	[9]

## Data Availability

Not Applicable.
